# Human Resource Management Challenges to Develop Pharmaceutical Industry: Evidence from Developing Countries

**Published:** 2018

**Authors:** Jafar Babapour, Arian Gholipourb, Gholamhossein Mehralian

**Affiliations:** a *Department of Pharmacoeconomics and Pharma Management, School of Pharmacy, Shahid Beheshti University of Medical Sciences, Tehran, Iran. *; b *Department of Human Resource Management, School of Management, Tehran University, Tehran, Iran.*

**Keywords:** Human resource management, Ability, Motivation, Opportunity, AMO model, Thematic Analysis

## Abstract

Human resource management has increasingly become one of the most important challenging issues in the pharmaceutical industry in general and in developing countries in particular to increase the access of societies to needed medicines. In this study, an attempt was made to explore the challenges of human resource management practices surrounding pharmaceutical firms in Iran, as a developing country. To answer the research question, a qualitative descriptive study using thematic analysis was performed through 22 semi-structured interviews with the key informants of Iranian pharmaceutical industry. Extracted themes of interviews were categorized into three main groups namely ability, motivation, and opportunity challenges based on the AMO model and briefly discussed separately. This is the first study investigating HRM challenges in the pharmaceutical industry; the research contributes to developing knowledge of human resource practices and assist pharma managers with better understanding how well implement human resource practices.

## Introduction

In the present knowledge-based economy, firms attempt to attain organizational goals with optimal exploitation of their resources. Humans, as a vital resource for organizations, not only have a decisive role in the evolution of firms, but also act as the leader of all other resources to conquer rivals in today’s dynamic atmosphere of businesses. Playing a pivotal role in success of the firms, human resource management (HRM) needs human resource (HR) systems, HR policies, and HR practices. There are obvious differences among these concepts. HR practices reflect specific organizational actions designed to achieve some specific outcomes. HR policies reflect an employee-focused program that influences the choice of HR practices. An HR system operates at an even higher level of analysis and reflects a program of multiple HR policies that are espoused to be internally consistent and reinforcing to achieve some overarching results (1). Human resource management practices (HRMPs) are managerial tools which are applied to form employees’ knowledge, skills, attitudes, and behaviors (2, 3). In other words, such practices involve organizational efforts to elicit discretionary activities from the workers (4, 5). These definitions properly reveal the potential effects of HRMPs on different parts of organizations. In this regard, over the recent decades, research on HRMPs and ultimate consequences of such practices (that are represented as organizational outcomes) have been more appealing to academics and practitioners. To further illuminate how this relationship occurs, some investigators proposed varying mechanisms known as ‘black boxes’ in the relationship between HRMPs and organizational performance (6). Therefore, to create repositories of knowledge and competencies in a firm, HRMPs are the central drivers and play an irrefutable function, especially in knowledge-driven fields. Moreover, it seems that proper recognition of HRMPs is crucial to understand knowledge-based views, human capital theory, and resource-based views, and that sufficient attention to the mentioned practices is necessary for firms to attain sustainable competitive advantage through their HRM (2, 6–13). 

Despite the wide range of studies focused on the outcomes of HRMPs, there are other studies believing that such practices are influenced by such factors as culture, climate, institution, business structure, and HRM professionals (14). Given the important role of ‘context-specific nature of HRM’, as pointed by Budhwar and Debrah, prior to the implementation of HRMPs, all managers should deeply analyze their organizational atmospheres in an appropriate manner; they further declared that, as the majority of the HRM studies have been conducted in western, developed nations, it is better to extend such studies in developing countries with their particular socio-cultural context (15). Along with the mentioned concern, there are some other issues that need to be addressed to fill the existing gaps. For example, some researchers have recommended evaluating pure effects of each practice on organizational performance to determine the value of all practices (16, 17). Upon these concerns, it is believed that studying challenges, obstacles and difficulties of each HRMP implementation would be beneficial to maximize organizational benefits, added value, and goal achievement. It is also believed that multilateral identification of HRMPs not only helps academics thoroughly define and precisely realize the practices, but also assists managers and practitioners in resolving their administrative problems and removing implementation barriers. 

Relying on its ancient cultural heritage, Iran possesses traditional HRMPs and policies. The country undergoes a transition phase toward organizational efficiency and adoption of advanced HRM systems (18). It is obvious that identifying HRMPs and recognizing their implementation challenges are theoretically and practically recommended for such a country. Furthermore, since goals, sizes, responsibilities, and resources of organizations vary from each other; therefore, the HRM specifications and related challenges in each business differ from one another. In other words, an organization working in a given industry like pharmaceutical one, have its specific HRM particulars and challenges. The nature of pharmaceutical industry and its technology-based as well as knowledge-driven environment make the industry a great candidate for this study (6, 19). In addition, this industry is intensively dependent upon its resources, especially human resources (HRs), to protect its sustainable competitiveness (20). Similar to other pharmaceutical companies around the world, pharmaceutical firms operating in Iran suffer from HRM problems such as retaining star scientists, talent management, training as well as development challenges, and so on (21). Indeed, the aforementioned challenges are not specific to Iranian context, but rather are very common in the current complicated relationships among managers and workers. However, only a few studies have been performed in this scope, and the present research is willing to contribute to the development of HR practices’ knowledge and assist managers of firms with better understanding of the gaps of HR practices. The literature covering HRMPs challenges in developing countries in general and in Iran in particular is dearth; hence, this lack of evidence encouraged us to study HRMPs challenges and to find how to deal with them. Therefore, the present study is aimed at addressing, through a qualitative study, the question of main challenges faced by HRMPs in pharmaceutical industry. 

The rest of this study is organized as follows; first, a review on existing literature is outlined; then, the research methodology is presented, followed by presenting the results, discussion, conclusion, and managerial implications, respectively.

*Theoretical background*

Based upon the theory of resource-based view, a large number of studies have paid great deals of attention to this theory in which it is believed that, among the entire spectrum of intangible assets available in a firm, human capital is the most important resource to achieve organizational performance and less competitive than other intangible assets. This issue is particularly pronounced in knowledge-based contexts such as pharmaceutical industry where people are well acknowledged as players of a pivotal role in gaining competitive advantage through creating new ideas and solving problems. In HRM literature, numerous investigations have been performed to understand the impact of HRMPs on both individual and organizational performance, and researchers have also strived to recommend that HRMPs would result in desired organizational outcomes. Therefore, the implementation methods of HRMPs performed by firms comprise a determinant part of the organizational success; hence, quite a few scholars have tried to underscore how HRMPs would be effectively implemented and under which mechanism such practices would affect organizational goals. For example, de Beeck *et al*. identified a black box called ‘HRM implementation by line managers’ that was considered as a gap between intended and implemented HR practices (22). This study resumed the efforts made by prior researchers such as Gratton and Truss who made clear organizations’ inability to achieve prosperity despite their best efforts in HRM activities (23). Also, Khilji and Wang found significant differences between the intended practices and the corresponding operationalized forms in organizations; they referred to the difference as a missing linchpin in strategic HRM and stated that the circumstances under which HRMPs are implemented would be considered as a distinguishing factor between traditional and non-traditional organizations. They also proposed that mere imitation of HRM is inadequate for creating value (24). Later on, Guest *et al.* developed a four-stage process of HRM implementation and made clear the role of line managers (25). 

Recent studies conducted during the last decade have put a great emphasis on how HRM implementation approaches would be effective and have also made great endeavor to move the cornerstone of HRM from HR department to other levels of organizations. For example, Woodrow and Guest stated that “high-quality HR practices count for little if they are poorly implemented” (26). Also, Trullen *et al.* declared that, a HR practice is effectively implemented when there is an ideal overlap between intended and actual practices (27). Following these pieces of research, Bondarouk *et al.* proposed conceptualizing and discovering successful HRM implementations based on contributions from different actors (28). In this view, HR decision makers such as top managers, HR professionals, and line managers as well as employees have their own tasks for performing the HRM process successfully in firms, so that identifying their duties and responsibilities would provide HRM with good governance. Generally, it seems that identification of HRM challenges not only addresses HRM inefficiencies, but also specifies the interventions that must be applied. It is evident that such studies would be critical and inevitable. For instance, in the case of HRMPs implementation, since the ways through which line managers coordinate their subordinates are a big concern to HR department in order to have HRMPs implemented effectively, and obstacles against the implementation of such practices need to be identified using line managers’ views. 

Accordingly, it is believed that it should be fruitful to analyze HRM challenges carefully and explore problems, barriers, and obstacles surrounding organizations. Such a structured evaluation has invaluable benefits for every stakeholder of the organization and minimizes the waste of resources. Despite the fact that HRM challenges are of great importance, only a small deal of attention has been paid to the issue, and detailed case studies on the analysis of particular practices have been performed just in recent years (26). In other words, as mentioned at the beginning of this section, a historical overview to the HRM literature in recent decades reveals that, investigations have been often focused on bundle-oriented approach rather than practice-oriented perspective (29); of course, in this kind of papers, some challenges and obstacles of HRM were occasionally discussed on demand. In this direction, it has been found that application of the methods adopted in individual case studies to investigate the factors related to HRM implementation can be very beneficial. It mainly helps clarify the causes and effects of imperfect implementation of HRMPs and the influence of the context on different HRMPs (26). Therefore, focusing on the practices and putting more emphasis on challenges raised by HRMPs in pharmaceutical industry of Iran, as a case study, this research investigates the HRM obstacles through interviewing, which is a common method in many studies on HRM challenges.

There are a wide range of approaches to HRMPs in the literature: universalistic, contingency, and configurational perspectives (30). In this study, our findings were based on AMO model. This model of HRM proposes that, employees’ performance is a function of three fundamental components consisting of their ability, motivation, and opportunity to perform (31). Although this model can be rooted back in the literatures of late 20^th^ century, Lepak *et al.* stated that, it might be useful for categorizing HR practices into either of three primary dimensions: skill-enhancing HR practices, motivation-enhancing HR practices, and opportunity-enhancing HR practices (1). To test this approach, several empirical studies analyzed this conceptual framework, well confirming its validity (32). In contrary to such systems as high-performance work practices (17), innovative human resource practices (33), and practices of successful organizations (34) that focus on a limited number of HR practices, this model covers more HRMPs applied in organizations while recognizing the HRMPs independently (31). It seems that adopting AMO model will be beneficial to design a study particularly for those who are trying to find a proper approach. Hence, this model is adopted to address the research questions properly in this research.

## Methods

As mentioned in the previous section, the aim of this study is to recognize the challenges of HRMPs in the pharmaceutical industry. The qualitative methodology implemented in this study involves the following steps:

*Choosing analytical approach*

To answer the study question, we needed to understand opinions, experiences, perceptions, and attitudes of the individuals who had actually confronted the challenges and obstacles of HRMPs in Iranian pharmaceutical firms. To this end, a qualitative descriptive approach has been recommended to help researchers acquire the specific phenomenon they pursue (35). For this purpose, thematic analysis defined as “a method for identifying, analyzing and reporting patterns (themes) within data” (36) seemed to be appropriate for this study; accordingly, semi-structured interviews were undertaken to gather required data for the purpose of the qualitative technique (37, 38). 

*Data gathering*

The required data in this study were collected via interviews. To develop a framework for the interviews, authors held a meeting to discuss around the interviewees’ eligibility, reviewing the main research questions, and the topics that had to be covered in the interview process. Since the researchers tend to investigate the topic of the study deeply and understand the answers thoroughly, a semi-structured interview was arranged. Restated, the main question in this study was ‘the challenges of HRMPs in the pharmaceutical industry’. Hints about challenges of well-known HRMPs such as staffing as well as selection challenges, training as well as development challenges, compensation as well as benefits challenges, performance management challenges, and succession planning challenges were provided whenever needed. In addition, before arranging interviews for gathering data, this study was approved by the Ethics committee of Shahid Beheshti University of Medical Sciences.

In order to identify appropriate participants as potential sources of knowledge or information, the researchers purposively arranged a list of candidates with adequate deals of knowledge and experience, including members of board of company directors, managing directors, chief executive officers (CEOs), HR managers, and academicians. It must be noted that even though the study researchers had arranged a list of 15 candidates of interviewees, the method used to gather data was purposive sampling followed by snowball sampling. In this manner, 22 interviews performed that 8 of them were not in the initial list and their selection was based on the judgment of interviewer, the situation of the study, and the suggestions of the interviewees. Each interview was performed within a pre-arranged time, mostly in the participant’s office. The interviewer was one of the authors who had adequate knowledge of HRM. The interviews were performed during January-March 2017. Since semi-structured interviews often contain open-ended questions, and discussions may diverge from the interview guide, it is generally best to tape-record the interviews and then transcribed them for analysis.

All interviews were recorded upon the interviewee’s permission. In some cases, because of the ambiguity and uncertainty of the recorded voice, the interviewee was recalled to clear up the subject. Upon accomplishment of the 22^nd^ interview, the researchers got into theoretical saturation and ended the interviews. As for the details on the interviews, it is be noted that mean (standard deviation) of interviewees experience (year), duration of interviews (minute), and number of the themes extracted from each interview are respectively 19.1 (12.2), 76.8 (22.4), and 20.3 (5.9). Also, about the educational degree of the interviewees, five had Ph.D, six had pharm. D, nine had M.A, and two of them had B.A. 

*Data analysis process*

Once finished with the interviews and their transcription, two of the researchers began to generate initial codes and cross checked them according to the method proposed by Braun and Clarke (36). In order to facilitate the codifying process, MAXQDA 10 software was utilized. Given the deductive approach of the present study, the researchers sought for a suitable model for categorizing the themes. Restated, considering the comprehensiveness of the themes extracted from the interviews, AMO model seemed to be an appropriate one with the capability to acquire all of the defined themes. As mentioned earlier, based on the AMO model, HR practices are categorized into either of three primary dimensions (1, 31). It is necessary to explain that in addition to deductive approach, some themes were inductively categorized in a class named “Miscellaneous challenges”.

## Results

As it was mentioned in the prior section, the results of this study were depicted based on AMO model. Accordingly, the recognized challenges of HRMPs, which are summarized in Tables 1-3, are explained in this section.

**Ability challenges**

*Staffing challenges*

In our study, three items were identified as the main obstacles for effective recruitment and staffing.

*Unskilled and unqualified manpower*

The interviewees pointed out three main defects, as shown in Table 1. One of the participants stated that “in general, educational systems should be able to match graduates capabilities with the needs of industries such as pharmaceutical industry; otherwise, finding qualified workforce for critical positions would become difficult”. Generic pharmaceutical producers are mostly involved in production and operation affairs; as CEO of a pharmaceutical company in Iran mentioned “university graduates often obtain theoretical capabilities rather than operational and executive ones, indicating that, educational centers are not well prepared to transfer work capabilities to workforce”. Moreover, the interviewees highlighted that pharmacists who serve as senior managers are often times suffering from lack of managerial capabilities; for example, one of the interviewees stated that, “pharmacists are taught pharmacology, toxicology, medicinal chemistry, *etc*., and do not learn enough about the principles of business and management sciences”. In addition, a manager of HR department of a pharmaceutical firm stated that “employees with master or Ph.D. degrees are appointed to managerial positions shortly after they are employed, while they are not well familiar with management principles and essential skills needed for managing an organization”.

*Inefficient staffing process*

All interviewees put emphasis on the undeniable role of staffing as well as selection procedures, and believed that such procedures are critical to assure the quality of HRs. They also mentioned that ‘formal written HR or staffing plans’, ‘tests administered prior to hiring’, ‘validated selection tests’, ‘different recruiting sources’, ‘rigorous selection techniques’, ‘extensive attention to capabilities’, and ‘assessing workforce’s knowledge and experience’ are the main concerns that play fundamental roles in HRM in companies. One of the participants stated that, “nepotism plays a key role in hiring new employees, particularly in state sectors”. 

*Inadequate professional candidates*

One of the well-experienced participants stated that, “generally, the industry does not provide interests for pharmacists; moreover, they are less paid and compensated compared to their responsibilities. Most pharmacists in Iran are interested in working in private and public pharmacies. It seems that pharmaceutical industry is not able to compete with retail pharmacies in recruiting pharmacists. Therefore, it is too difficult to find experienced pharmacists to hire”. 

Another interviewee mentioned: “considering the undeniable role played by pharmacists in the pharmaceutical industry, one of the most important incentives for pharmacists to step in the industry is to attain managerial and key positions in a firm, resulting in such positions being less available for other professionals such as chemists who have a prominent role in the industry all over the world”.

**Training and development challenges**

*Inefficient training of human resources*

The interviewees stated several factors resulting in deficiency of training programs on generic pharmaceutical producers in Iran, as reported in Table 1. Moreover, they mentioned that some companies fail to offer appropriate plans for training their employees for reasons such as ‘not having a clear goal for training’, ‘not identifying real training needs’, ‘providing theoretical trainings rather than applicable ones’, and ‘lack of attention to problem solving trainings and team building plans’. 

Most of the interviewees asserted that due to legal requirements, the pharmaceutical companies do their best to develop firm-specific knowledge and skills, but they do not pay adequate deal of attention to non-technical skills such as professional ethics, organizational values, negotiation skills, inter-personal relations, and behavioral skills. For instance, one of the participants stated that, “in some organizations, training is not considered as a priority; and failure to have an appropriate attitude towards training have caused managers consider training more of as a cost rather than an investment”.

*Inattentiveness to the development of human resources*

Participants believed that HRs development has been widely ignored as only few companies have addressed that. One of the interviewees said “development discussions have just recently been started in our company. To do so, we identified talents in the company and work on their skills; for example, some mentors have been hired to teach our managers, coaches, and middle-managers on how to behave the staff. To this end, our top managers need to be taught too”.

**Talent management challenges**

*Incapability in the retention of talented people*

Retaining knowledge workers is one of or even the principle of HRM challenges in public companies. “Totally, a little has been done for managing our talents in pharmaceutical companies, and consequently, our talents and knowledge workers have always been hunted by competitors; as a result, average turnover rate of capable and talented employees in our organizations is high”, one of our participants said. It is also reported that, payment is not the only reason for the workforce turnover, as some quit the company either because of conflicts with coworkers and managers or since they receive offers from other companies. 

*Inattentiveness to talent management and succession planning*

 One of HR managers interviewed in this study stated that, “only a few Iranian pharmaceutical companies deploy systematic and scientific succession plans; we are trying to utilize such plans through job rotations and training courses as well as developmental programs. These policies are not merely offered for managers and CEOs, but include all workers”.

**Motivation challenges**

In this section, following the ability challenges, the findings pertaining to motivation challenges are presented in three categories, as shown in Table 2.

**Compensation challenges**

*Inefficiency of compensation systems*

One of the main factors contributing to compensation challenges is inefficiency of HR grading system. In most of Iranian companies, job grading tools are based upon Iranian National Occupational Classification System. One of the interviewees believes that “the system is not efficient anymore as it works for conventional companies and cannot be applied for knowledge-based ones and research centers”. One of the participants stated that “in knowledge-based organizations like pharmaceutical companies, the co-working of low-skilled workers with knowledge staff in research and development activities centers in one hand, and the managers’ personal views on compensation systems along with legal limitations on payment systems, on the other hand, have ended up with dissatisfied employees feeling a sense of inequity across the company”. 

In addition, considering job opportunities available for pharmacists, one of the interviewees mentioned that, “pharmaceutical companies have to compete with community pharmacies in employing pharmacists; hence, it is undeniable that the pharmacists prefer to work in pharmacies where they work less and are paid more compared to the industry”. It is also obvious that the diversification of compensation schemes is an important function; for example, one of the HR mangers stated that “in order to optimize the effectiveness of compensation, it is crucial to identify that, key positions deserve compensation prior to compensate other employees; otherwise, employees may develop a sense of discrimination”. 

*Low salary base*

One of HR managers stated that “payments rate is increasing at lower rate than the inflation rate, and generable revenue by the industry is limited and controlled by the strict pricing policies set forth by the government, leading to dissatisfaction of the manpower”.

*Lack of assured salary data bank*

One of the participants mentioned that, “due to the deficiency of the grading system in majority of companies, lack of data banks containing salary surveys data has reduced our power against high salary requests by some experts; this leads to make a decision in a gloomy condition”.

**Performance management challenges**

*Unstructured performance management*

A senior manager of a pharmaceutical company believed that “in order to achieve organizational goals, besides improving organizational culture and creating the capabilities necessary in every workforce to be appraised, valid indicators and tools should be applied, and the evaluators have to be trained regularly”.

*Lack of linkage between performance appraisals and compensation, training opportunity, and promotion*

“In Iranian pharmaceutical industry, performance management systems are not fully implemented; the reason behind this issue is that for implementing a performance management system, some infrastructures are needed”, one of the interviewees stated and continued his talk, “the methods used to appraise employees’ performance and link the benefits to their evaluated performance are highly recommended as allocating resources without measuring the employees’ real performance may end up creating an improper right for them, as a result of which, the employees will think that there is no rational association between their received benefits and appraisals”. 

**Promotion challenges**

An experienced HR manager believes that “if a company fails to offer its workforce a positive prospect about their job promotion in the future, it will neither receive their optimum cooperation nor be able to maintain them for long time. Albeit, the promotion activities should be based upon the employees’ capabilities and development”. 

*Seniority versus merits*

“Taking into account the seniority of employees instead of their capabilities when it comes to offering key positions in the course of promotion process would mislead us to a wrong decision”, one of the participants said. 

*Lack of clear career path*

Developing a clear career path is necessary in every organization. In this regard, one of participants detailed that “lack of clear promotion guidelines reduces active participation of workforce, stifles their creativity, causes job exhaustion, and finally makes talented workforce leave the company”.

**Opportunity Challenges**

In the following, finding of the opportunity challenges are presented in two categories, as shown in Table 3.

**Communication and information sharing challenges**

*Lack of information and knowledge sharing*

Given the importance of knowledge sharing behavior, one of interviewees stated that, “unfortunately, some senior managers do not pay enough attention to the information and knowledge sharing, while by doing so, companies would be able to develop and internalize employees’ know-how in the firm”. In addition, high turnover of top managers is another concern of HRM in the Iranian pharmaceutical industry where a large number of companies are state-owned. In this line, one of HR mangers mentioned that “as the managers working in the pharmaceutical industry are changed repeatedly, their priceless knowledge and experience would be missed once they leave the company. For this reason, companies should not only pay especial attention to knowledge sharing, but also invest in the documentation of their organizations’ routines, patterns, and current practices”.

*Communicational obstacles*

According to interviewees, there are several communication challenges in Iranian pharmaceutical firms such as ‘interpersonal relations’, ‘communicating with superiors as well as sub-ordinates’, ‘inter-organizational relations’, and ‘ cross-functional relations’. For instance, one of the participants who were CEO at a pharmaceutical company mentioned that, “I gained surprising results through communicating with universities and hired talented graduates who have brought us considerable deals of added value”. 

**Insufficient attention to empowerment factors**

*Inattentiveness to the empowerment* *strategies for employees*

“Paying enough attention to the strategies aimed at empowering the employees would flourish the potentials of workforce for achieving organizational goals and create opportunities to identify talents who can subsequently solve problems and make right decisions. By taking this into account, a company is more likely to be successful in terms of succession plan and retaining knowledge-workers”, one of the employees stated.

*Inability to collaborate*

One of the interviewees mentioned that, “even though there are some diverse plans in companies to build working groups for the purpose of problem solving and involving others in decision-making process, such plans often fail to work in practice and end up with no higher organizational performance. Moreover, there is neither organized system for posting suggestions nor a formal grievance procedure and/or complaint process in most of Iranian pharmaceutical firms”.

**Miscellaneous challenges**

Beside the mentioned challenges categorized based on AMO model, some issues were revealed by the participants during the course of interviews. In this section, brief explanations of these issues are outlined.

**Imperfective HRM governance**

The interviewees referred to some main factors contributing to imperfective HRM governance in the companies. These included ‘traditional approach to HRM’, ‘insufficient attention to HRM strategy’, and ‘non-advanced views of top managers on HRM’. In this line, one of the participants argued that “senior managers’ non-technical view on HRM leads to hiring unskilled HR managers; in such a case, the HRM department is administered by a person who is not well-qualified in the field, resulting in different HR problems”.

**Organizational culture challenges**

In this relation, our interviewees mentioned three critical challenges: ‘change management’, ‘conflict management’, and ‘presence of different generations’. 

Change and conflict managements are long known challenges in organizations. One of the interviewees said “as the main body of an organization is made up of people with great deals of experience along with those who are newly hired, top managers are required to be familiar with the change and conflict management concepts as such skills enable managers to orchestrate employees with different demographic profiles”.

## Discussion

Given the crucial role of HRs in knowledge-based industries, an attempt was made to identify HRMPs challenges surrounding pharmaceutical managers in a developing country such as Iran. To this purpose, a qualitative study using thematic analysis was performed through inviting key informants of the industry. In Section 4 on findings, the participants’ quotations on HRM challenges were briefly reviewed. In this section, we focused on the themes to which the interviewees referred more frequently.

One of the most important challenges surrounding Iranian pharmaceutical firms, which have shed light on the entire body of HRM functions, is the traditional view on HRM; this view considers workforce more of cost rather than investment, adversely affecting the employees’ attitude and preventing their development. Some of the interviewees believed that in their companies, unfortunately, HRM was not understood well and paid enough attention to. This is a fundamental problem which has gained attention from researchers since decades ago (39). In order to deal with this issue, it is necessary to change traditional HRM approaches to strategic thoughts (15).

**Table 1:** Summary of ability challenges stated by the study participants

**Items**		
Staffing challenges	Unskilled and unqualified manpower	Inefficient managerial capability of workforce (19*)
		universities' insufficiency to graduating professionals to the industry (12)
		Insufficient ability of workforce (5)
	Inefficient staffing processes	Lack of comprehensive, rigorous, and validated employment testing (20)
		Employment not based on meritoriously (8)
		Inefficient recruitment procedures (agencies, …) (2)
	Inadequate professional candidates	Scarcity of experienced pharmacists (3)
		Pharmacist's little attraction to the industry (3)
		Inadequate non pharmacist experts stimulation to entry to the industry (3)
Training and development challenges	Inefficient training of human resource	Low effectiveness of training activities (8)
		Inadequate firm-external training activities (7)
		Carelessness to the quality of training activities (7)
		Carelessness to the non-firm-specific abilities' training activities (6)
		Out of date perception and unplanned training programs (5)
		Training activities' supervisory is not by the HRM department (3)
	Unstructured training activities	Imperfect training needs assessment (7)
		Inequality of training distribution (3)
	Inattentiveness to the development of human resource (6)	
Talent management challenges	Incapability in the retention of talented people (20)	
	Inattentiveness to talent management and succession planning (16)	

* Numeric indicates to the numbers of participants referring to the item.

**Table 2:** Summary of motivation challenges stated by the study participants

**Items**	
compensation challenges	Inefficiency of compensation systems (20*)
	Low base salaries (10)
	Lack of assured salary data bank (4)
Performance management challenges	Unstructured Performance management (14)
	Lack of linkage between performance appraisals and compensation, training opportunity and promotion (10)
Promotion challenges	Seniority versus merits (7)
	Lack of clear career path (6)

* Numeric indicates to the numbers of participants referring to the item.

**Table 3:** Summary of opportunity challenges stated by the study participants

**Items**	
Communication and Information sharing challenges	Lack of information and knowledge sharing program (9*)
	Communicational obstacles (6)
Insufficient attention to empowerment factors	Inattentiveness to the empowerment strategies for employees (6)
	Inability to collaborate (6)

* Numeric indicates to the numbers of participants referring to the item.

Paying inadequate attention to the staffing and selection practices was among the challenges more emphasized in this study, which relates to several distinct domains. First of all, lack of comprehensive, rigorous, and validated hiring tests in one hand, and recruiting not based on meritoriousness on the other hand, are defined as the two main challenges in staffing processes, especially in public sector. Hence, under these circumstances, selecting talents, who play a critical role in the pharmaceutical industry, is unlikely to achieve (32). Second, the presence of the educational gap between universities and the industry results in dealing with unskilled and unqualified workforce. To tackle this issue, it seems that universities should offer programs aligned with the needs of industry and make an effort to improve graduates’ understanding of the potentials of the industry in offering job opportunities (40). In addition to the reasons mentioned above, there are other issues deterring workforce from seeking job in the industry such as lower income compared to other positions and existing discriminations in promotion systems; this forces mangers to hire unqualified candidates over time.

As mentioned earlier, according to the participants’ views, succession planning and talent management are two challenging issues in organizations; talents comprise the biggest capital of knowledge-based companies. Due to high costs of research and development in pharmaceutical companies, succession in competitive markets depends highly on the way talented staffs are retained in the firms (21). In addition, there is a vast range of literature highlighting the way through which companies can retain talents and approach to succession planning (41, 42). Therefore, mangers in the industry should find ways to manage talents and remove succession barriers such as lack of proper culture, fear of losing positions, and employees’ retention uncertainty. Our findings about lack of succession plans are in agreement with a recent study performed in Iranian health sector (43). 

Training and development are of the main functions of HRM which enrich the workforce’s pool of knowledge, improve human capital, and ultimately build valuable intangible assets (6). Therefore, inattentiveness to the learning power in the workplace and inefficient training as well as development programs weaken an organization’s power to survive in uncertain conditions (44). 

Given the important role of medicines in providing societies with health, there are strict regulatory controls and requirements in the pharmaceutical industry (or medicinal production procedures) in almost all countries; consequently, technical education standards are developed to meet the requirements (45). Commonly, a technical manager is responsible of fulfilling these requirements; however, appointing a technical manager who might not be familiar with the HRM approaches results in focusing on technical pharmaceutical-oriented trainings only, rather than non-technical ones. Furthermore, in most Iranian companies, majority of the training programs offered to the staff are using existed potentials inside the organization instead of using external resources, while in such a strongly competitive industry, managers have to be sufficiently trained about such skills as contracting, negotiation, commercialization, to name a few (19). 

Besides the challenges discussed earlier, some others would reduce employees’ motivation, and one of them is deficiency of compensation packages. Undoubtedly, compensation system is one of the major HRM functions which contribute largely to the success of companies (46, 47). Findings indicated that the compensation systems suffer from critical obstacles rooted back in the economic conditions of companies which deprive employees of fair compensation packages. On the other hand, lees attention to various reward management strategies in the organization results in the inefficient punishment and encouragement procedures which impede the motivation of the employees. 

Consistent with other investigations, our study strongly recommended performance appraisals as major HRMPs to alter employees’ behaviors (48, 49) and achieve innovation performance (50) It is perceived that most companies lack infrastructures required for implementing performance management systems and such systems are not well institutionalized in the companies. This might stem from some conditions in organizations such as ‘unfamiliarity of mangers with appraisal’, ‘weak appropriate feedback’, ‘refusal by subordinates’, and ‘unfair, unrealistic as well as inaccuracy appraisals’. Additionally, our results confirmed the findings of previous studies, indicating that in Iran, workforce would be promoted mostly based on seniority rather than merits (18). It is largely believed that failing to pay adequate attention to meritocracy and a promotion system would weaken dynamism and keep workforce unproductive.

While it is well-acknowledged that information sharing, empowerment factors, and collaborations can significantly contribute to the innovation capabilities and organizational performance (33, 51, and 52), our findings demonstrated that such factors were not well approached in the industry. This is mainly because of lack of knowledge management systems (KMS) in storing, sharing, and utilizing the existing knowledge, while such systems are greatly emphasized by scholars in order to harmonize knowledge activities across a company (50, 53). One of the ultimate goals of KM is to share knowledge among knowledge workers who play critical role in a knowledge-intensive industry like pharmaceutical one (54).

Other than HRMPs’ challenges identified based on AMO model, there were some other issues highlighted by the participants such as change management, conflict management, and differences between generations. 

As an example of these three, Cogin pointed out that managing the conflicts between younger and older workers has recently become one of the most important challenging issues as the perceptions of work ethics and work-life balance requirements are quite different between them (55); this was particularly stated by the participants in our study who proposed that in order to address this issue, HR managers have to adopt scientific and acclaimed mechanisms to manage these two generations in a today’s complex environment. the Management of the mentioned issues plays a critical role in the organizational performance. For instance, like other studies, we argue that using HRMPs to improve employees’ abilities to deal with organizational changes through high participation in decision-making, high-quality teamwork, and high autonomy will improve proactivity of the organizations (53).

Finally, based on the results of this research, the nature of the Iranian pharmaceutical industry has some specific characteristics that influence the functions of HRM: ‘capital structure of Iranian pharmaceutical companies’, ‘generic regimes’, ‘market size’, and ‘pricing systems’. It seems that as the capital structure of the most Iranian pharmaceutical companies is state-owned, there would be too difficult to compete in a quite free market, resulting in forming a generic market and highly restricted pricing systems. We believe that although these factors are specific to our research context, they potentially influence HRM practices of all other industries. 

## Conclusion

In this study, an attempt was made to use thematic analysis to explore HRMPs challenges surrounding pharmaceutical firms in Iran as a developing country. Our findings were categorized into three main groups based on AMO (ability, motivation, and opportunity) model. With respect to the ability, staffing challenges, training as well as development challenges, and talent management challenges were the three themes identified. Regarding the motivation, the deficiency of compensation packages, inefficient performance management, and promotion challenges were emphasized by the participants. As far as the opportunity was concerned, two themes were generated: communication as well as information sharing challenges and insufficient attention to empowerment factors. In addition to the mentioned themes, some miscellaneous challenges were recognized, including the imperfective HRM governance, the organizational culture obstacles, and the nature of the industry.

This study revealed some HRMPs that should be taken into account in developing countries and highlighted barriers for institutionalizing such practices given socio-cultural setting in these countries. 

Finally, it can be concluded that being theoretically aware of HRM concepts, Iranian top managers are gradually trying to implement HRMPs in their organizations. 

Briefly, in the recent years, as it is reflected in the previous research, the Iranian organizations have begun to pay further attention to HRM, and to work on the development of people (56). It is also understood that the Iranian HRM has considerable similarities with those of other developing countries in terms of, for instance, rigid and hierarchical organizations, unplanned decision-making, ascription-based promotion, and lack of performance-orientation in compensation and appraisal (18, 57).

## Managerial implications

This study offers key implications for the top managers willing to improve their organizations’ effectiveness through HRs, especially in developing countries. In addition, the study satisfies the pharmaceutical industry practitioners undergoing intensive competition and endeavoring for sustainable competitiveness. In this regard, our study serves several important implications for managerial practice from an HRM viewpoint:

First, the members of board of company directors and other top managers must realize the fundamental role played by HRM in the success of their organizations and change their perception of a traditional HR to a strategic view.

Second, CEOs should consider their HR departments as the most important unit of the firm that can serve as a great, valuable source to achieve sustainable competitive advantage.

Third, HR managers must scrutinize their package of HRMPs consistent to the firms’ context and strategy and identify the barriers against HRMPs implementation in the firms, so as to adopt appropriate HRM systems to achieve organizational goals.

Fourth, if HR managers pay enough attention to the staffing and selection processes of their organizations and apply rigorous and validated hiring tests, they can be assured too much to their prosperity. In other words, the managers should consider adopting a meritocratic approach in their staffing processes to achieve organizational successfulness.

Fifth, the senior managers of the pharmaceutical industry should be aware of the importance of non-technical training programs and attempt to extend this kind of educations to different levels of the employees. In this regard, training of the skills such as problem solving, interpersonal relations, and negotiation techniques have been considered in this study.

Sixth, line managers should realize their undeniably critical role in the implementation of HRMPs in the firms and attempt to improve their knowledge about HRM so that they would be able to manage HR performance effectively.

Seventh, HR managers and other top managers of the organizations should be aware of different aspects of HRM and pay enough attention to all of its functions. For example, HR managers should pay sufficient attention to AMO attributes (*i.e. *ability, motivation, and opportunity) to maximize their contribution to the success of the firms.

Eighth, the results of this study shed light on practices that must be undertaken by managers, especially in the pharmaceutical sector, and the ways through which the managers can increase the benefits of investing in HRMPs in their organizations.
